# Supplementary material for: Expression of POU2F3 Transcription Factor Control Inflammation, Immunological Recruitment and Metastasis of Pancreatic Cancer in Mice
Source: Biology (Basel). 2020 Oct 19;9(10):341. doi: 10.3390/biology9100341 (PMC7603360; doi:10.3390/biology9100341)
Supplement: Supplementary file 1 [file biology-09-00341-s001.pdf]

Table S1. Antibodies.

Name		Brand	Reference	Clone	Condition	Dilution
CD117	m	Millipore	CBL1360		TRS6	1/100
CD11b	m	BD Pharmingen	553308	M1/70	TRS pH9	1/100
IL17AR (CD217)	h/r/m	ThermoFisher Scientific	PA5-34571		Citrate	1/200
CD4	m	Abcam	ab64144		TRS9	1/500
CD8a	m	BD Pharmingen	553027	53-6.7	TRS6	1/100
DCLK1	m/h	R&D	AF7138		Citrate	1/500
POU2F3	m	santa cruz	sc-330		Citrate	1/500
DCLK1	h/r/m	Abcam	Ab31704		Citrate	1/500
F4/80		santa cruz	sc-71085	6A545	Citrate	1/100
NOS2	m/r	Santa cruz	sc-650	M-19	Citrate	1/100

Table S2. Primers.

36B4	GCTGATGGGCAAGAACACCA	CD68	TGTCTGATCTTGCTAGGA
	CCCAAAGCCTGGAAGAAGGA		AGGAGAGTAACGGCCTTT
**IL-17A**	TCAGCGTGTCCAAACACTGAG	**CD44**	TCTGCCATCTAGCACTAAGAGC
	CGCCAAGGGAGTTAAAGACTT		GTCTGGGTATTGAAAGGTGTAGC
**Bcl-XL**	AGTGAAGCAAGCGCTGAGA	**CXCL1**	ACTGCACCCAAACCGAAG
	TCAGGAACCAGCGGTTGAAG		TGGGGACACCTTTTAGCA
**IL6**	CCGGAGAGGAGACTTCACAG	**IFNy**	ATGAACGCTACACACTGCATC
	GGAAATTGGGGTAGGAAGGA		CCATCCTTTTGCCAGTTCCTC
**IL-9**	ATGTTGGTGACATACATCCTTGC	**CXCL2**	CCAACCACCAGGCTACAGG
	TGACGGTGGATCATCCTTCAG		GCGTCACACTCAAGCTCTG
**IL-4**	CCCCAGCTAGTTGTCATCCTG	**CXCL5**	GTTCCATCTCGCCATTCATGC
	CAAGTGATTTTTGTCGCATCCG		GCGGCTATGACTGAGGAAGG
**Bax**	ATG CGT CCA CCA AGA AGC TGA G	**CXCR2**	ATGCCCTCTATTCTGCCAGAT
	CCC CAG TTG AAG TTG CCA TCA G		GTGCTCCGGTTGTATAAGATGAC
**MUC1**	GGCATTCGGGCTCCTTTCTT	**IL13**	GAG AGA GGA GTT GGC GTC AC
	TGGAGTGGTAGTCGATGCTAAG		TTC AAC AGG CTA AGG CCA CA
**IL-1b**	GTG GCT GTG GAG AAG CTG TG	**Gata3**	CTCGGCATTCGTACATGGAA
	GAA GGT CCA CGG GAA AGA CAC		GGATACCTCTGCACCGTAGC
**TNFa**	AGTCCGGGCAGGTCTACTTT	**RORgt**	CCGCTGAGAGGGCTTCAC
	AAGCAAAAGAGGAGGCAACA		TGCAGGAGTAGGCCACATTACA
**Bid**	CACGCCAAGGTCTTTCCAT	**T-bet**	GTATCCTGTTCCCAGCCGTTTC
	GTCAGGAACTTGGTTAGAAACGA		ACTGTGTTCCCGAGGTGTCC
**CD164**	GTG TTT CCT GTG TTA ATG CCA C	**IL13**	AGCTCCCTGGTTCTCTCACT
	CAC AAG TCA GTG CGG TTC AC		CTCATTAGAAGGGGCCGTGG
**IL10**	GCTCTTACTGACTGGCATGAG	**MMP19**	GGTGTTCCTGTTTAAGGGCTCA
	CGCAGCTCTAGGAGCATG		GCCATCTTGCCAGCTCATAG
**VIM**	CGGCTGCGAGAGAAATTGC	**N-cadhérine**	AGCGCAGTCTTACCGAAGG
	CCACTTTCCGTTCAAGGTCAAG		TCGCTGCTTTCATACTGAACTTT
**MUC1**	GGCATTCGGGCTCCTTTCTT	**E-cadherin**	CAGGTCTCCTCATGGCTTTGC
	TGGAGTGGTAGTCGATGCTAAG		CTTCCGAAAAGAAGGCTGTCC

Table S3. Primary antibodies for Flow Cytometer analysis.

Name	clone	Reference	Dye	Brand
CD45.2 BUV737	104	564880	BUV737	BD bioscience
CD11b BUV395	M1/70	563553	BUV395	BD bioscience
m MHC II (I-A/I-E) A700	M5/114.15.2	56-5321-82	Alexa 700	eBioscience
Ly6G BV421	1A8	562737	BV421	BD bioscience
m Ly-6C FITC	AL-21	553104	FITC	BD bioscience
CD64 BV711	X54-5/7	139311	BV711	Biolegend
F4/80 BV785	BM8	123141	BV786	Biolegend
SiglecF PECF594	E50-2440	562757	PE-CF594	BD bioscience
Tim4 APC	RM14-54	130008	A647	Biolegend
CD226 Pe-Cy7	IOE5	128811	PE-Cy7	Biolegend
CD124 PE	mIL4R-M1	552509	PE	BD bioscience
NK1-1 APC-Cy7	PK136	108724	APC-Cy7	Biolegend
CD19 APC-Cy7	1D3	561737	APC-Cy7	BD bioscience
CD3ε APC-Cy7	145-2C11	561042	APC-Cy7	BD bioscience
CD4 BV785	RM4-5	563727	BV786	BD bioscience
CD8α PeCy7	53-6.7	25-0081-82	PE-Cy7	eBioscience
CD279 (PD1) biot	RMP1-30	13-9981-82	biotin	ebioscience
SA PE		12-4317-87		ebioscience
CD273 APC	TY25	560086	APC	BD bioscience
CD117 PE	2B8	561075	PE	BD bioscience
FceR1 PeCy7	mars-01	25-5898-82	PE-Cy7	eBioscience
CD25 APC	PC61 Ac de rat	557192	APC	BD bioscience
CD127 biot	A7R34	13-1271-85	biotin	ebioscience
SA BV785		563858		BD Horizon
CD16/32 BV510	2.4G2	740111	BV510	BD
IL-33Ralpha (T1/ST2) BV421	DIH9	145309	BV421	BioLegend
Integrin β7 FITC	M293	09324A	FITC	BD bioscience
Ly6G A700	1A8	561236	Alexa 700	BD bioscience
m CD11b BV711	M1/70	101242	BV711	biolegend
CD279 (PD1) PeCy7	RMP1-30	109110	PE-Cy7	BD bioscience
m CD8β PE	H35-17.2	550798	PE	BD bioscience
m CD169 (Siglec-1) APC	SER-4	55-5755-80	eFluor660	eBioscience
Ly6C PECF594	AL-21	562728	PE-CF594	BD bioscience
SiglecF PE	ES22-10D8	130-102-274	PE	Miltenyi
m CD11c APC	N418	17-0114-81	APC	eBioscience
